# Keratinocytic epidermal nevus syndrome with Schwann cell proliferation, lipomatous tumour and mosaic *KRAS* mutation

**DOI:** 10.1186/s12881-015-0146-5

**Published:** 2015-02-10

**Authors:** Said Farschtschi, Victor-Felix Mautner, Silke Hollants, Christian Hagel, Marijke Spaepen, Christoph Schulte, Eric Legius, Hilde Brems

**Affiliations:** Department of Neurology, University Medical Center Hamburg-Eppendorf, Martinistrasse 52, 20246 Hamburg, Germany; Department of Human Genetics, KU Leuven – University of Leuven, Leuven, Belgium; Institute of Neuropathology, University Medical Center Hamburg-Eppendorf, Hamburg, Germany; Department of Human Genetics, University Hospital Leuven, Leuven, Belgium; Institute for Heamatopathology, Hamburg, Germany

**Keywords:** Keratinocytic epidermal nevus syndrome, *KRAS*, Mosaicism, RASopathy, Somatic mutation

## Abstract

**Background:**

Keratinocytic epidermal nevus syndrome (KENS) is a complex disorder not only characterized by the presence of epidermal nevi but also by abnormalities in the internal organ systems. A small number of cases with KENS are molecularly characterized and reported in the literature with somatic activating *RAS*, *FGFR3* and *PIK3CA* mutations.

**Case presentation:**

In this study we present a patient with hyper- and hypopigmented regions, verrucous pigmented skin lesions and a paravertebral conglomerate tumour at the level of the cervical and thoracic spine. A large lipomatous dumbbell tumour caused atrophy of the spinal cord with progressive paraparesis. We identified a mosaic c.35G > A (p.Gly12Asp) *KRAS* mutation in the pigmented verrucous epidermal nevus tissue, the intraneural schwann cells and the lipoma. The c.35G > A (p.Gly12Asp) *KRAS* mutation was absent in the peripheral blood leukocytes.

**Conclusion:**

We conclude that KENS, the intraneural Schwann cell proliferation and the lipoma in this individual were caused by a postzygotic and mosaic activating c.35G > A (p.Gly12Asp) *KRAS* mutation.

## Background

Ras proteins play a crucial role in cellular growth factor signaling. *RAS* mutations, found in 30% of human tumours, have an activating effect on the protein, are oncogenic and activate a number of downstream pathways [1].

Non-organoid keratinocytic epidermal nevus (KEN) is characterized by benign congenital hyperpigmented skin lesions following Blaschko’s lines. Epidermal nevi with localized epidermal thickening are present at birth or become visible during early childhood. KEN in association with extracutaneous lesions in brain, muscular skeleton or eye is defined as keratinocytic epidermal nevus syndrome (KENS). Neurological abnormalities can include seizures, cognitive impairment, developmental delay and hemiparesis. Potential skeletal abnormalities are abnormal curvature of the spine and incomplete bone formation.

Mosaic *FGFR3*, *PIK3CA* and *RAS* mutations are recognized in KEN with *HRAS* as the most prevalent mutated gene [2-4]. *RAS* mutations are reported in mosaic RASopathies i.e. non-organoid KEN, sebaceous nevus and in extra-cutaneous manifestations of the corresponding syndromes, KENS and Schimmelpenning syndrome [5,6].

Only a few patients with epidermal nevus syndrome are molecularly characterized. In the context of understanding the phenotype-genotype in RASopathies, especially in epidermal nevus, we describe a patient with hyperpigmentation and severe neuroskeletal abnormalities.

## Case presentation

The patient was born by Caesarean section (weight 3760 g, length 55 cm) and developed normal speech, while motor development was delayed (free sitting at 12 months, back to belly turn at 13 months, pulling into stand at 18 months). Within the first years of life, the patient developed pain in the left arm as well as progressive paraparesis. Surgical removal of compressive intraspinal, extramedullar components of a tumour was performed at the age of 4 years with tumour reduction and partial relief of symptoms.

**Figure 1:**
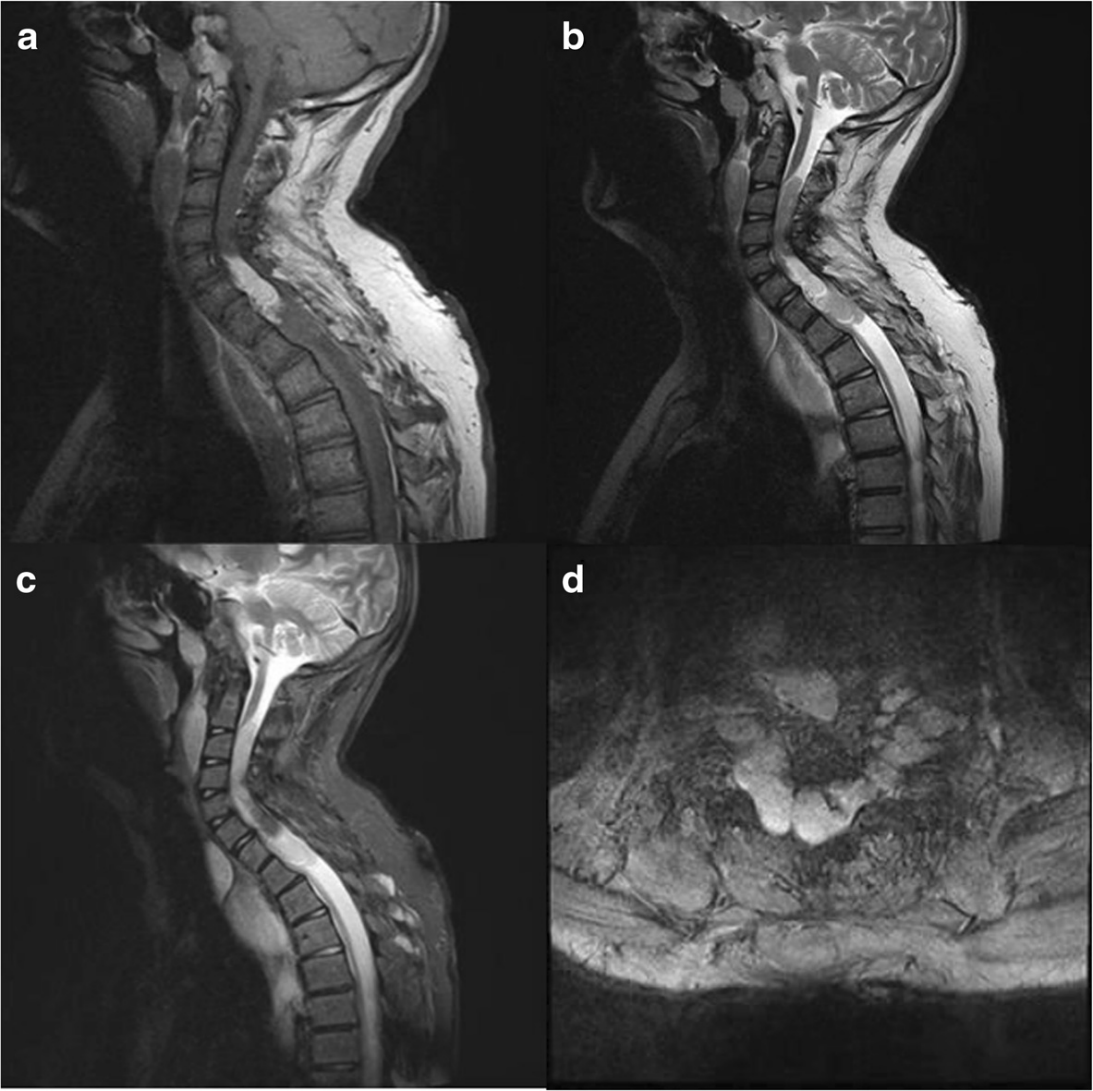
**3T-MRI of the upper spine: T1 weighted sagittal image showing a large cervical and thoracic tumour mass (a).** T2 weighted sequences **(b)** detect extensive intraspinal tumour formations, partially fat-isointense on fat-suppressed sequences **(c)**. Transversal T2-images show the dimensions of the paravertebral mass **(d)**.

At 14 and 16 years of age follow up MRI demonstrated a large paravertebral conglomerate tumour next to the cervical and thoracic spine with extensive additional intraspinal components (Figure 1a-d). Consecutively, there was severe narrowing of the spinal canal with spinal cord compression. The intraspinal tumour was a long cylindrical, inhomogeneously configured mass, which filled the spinal canal incompletely from C3 to T1. In addition, there was a remarkably large intraspinal lipoma located between C5 to C7, which showed typical bright pillow-like tissue (fat isointense on T1 weighted imaging) presenting dark in T2-weighted fat suppressed sequences. The spinal nerves (C3-T1) on both sides showed cylindrical or dumbbell-like thickening with intraspinal expansion, so that the spinal cord was compressed from the right side below C3. Consecutively, there was atrophy of the spinal cord below segment T2. Additionally a right convex thoracolumbar scoliosis was found. Cranial MRI showed no abnormalities. Ophthalmologic examination showed bilateral impaired vision (50%) and astigmatism with no Lisch nodules or other abnormalities.

**Figure 2:**
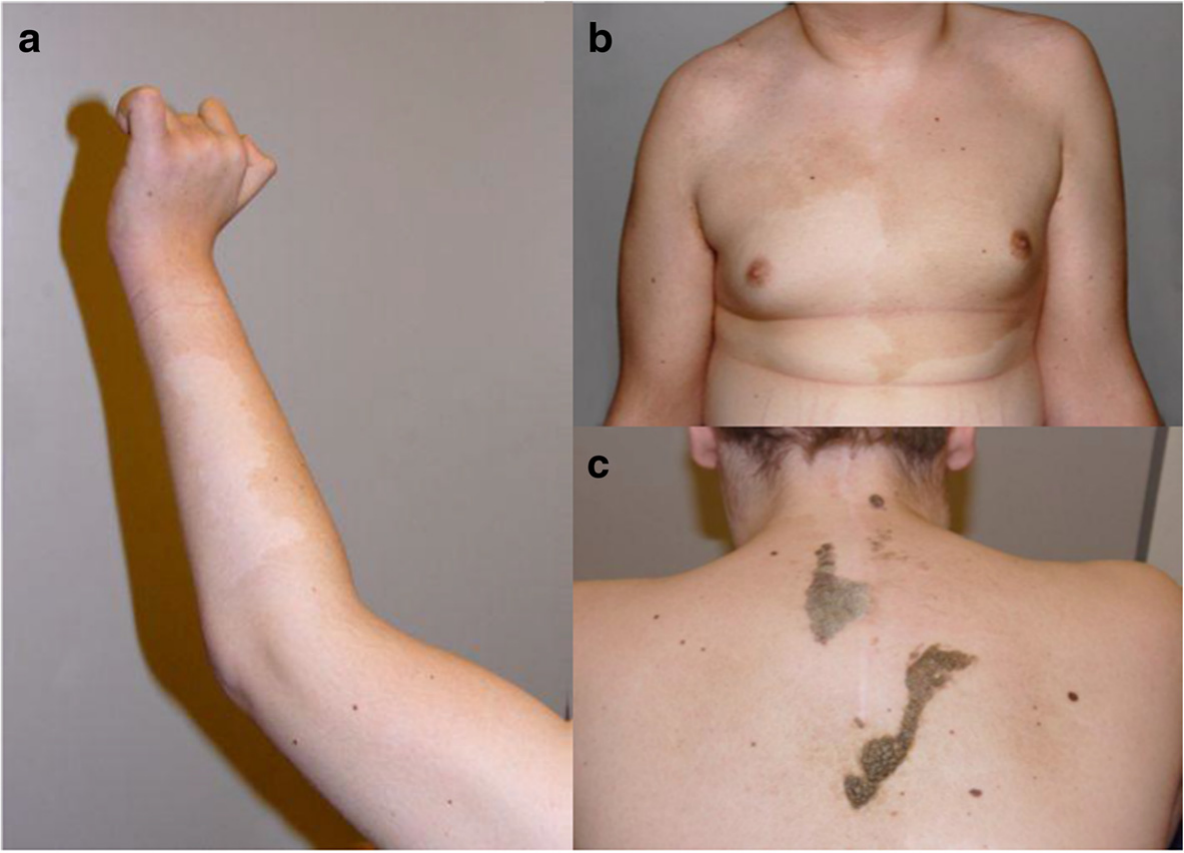
**Clinical findings of the skin by follow-up presentation: Large inhomogeneous hyper-and hypopigmentation of the upper extremities (a) and the trunk (b) with serrated and polycyclic borders.** Verruceal corneated mass on the upper back with tongue shaped tails and granular surface; median scar from precious neurosurgical intervention **(c)**.

At follow-up at 17 years of age he showed a large hyperpigmentation and hypopigmentation of upper body parts ranging from the trunk to the arms. There was partly polycyclic/polygonal hyperpigmentation of chest and back (Figure 2a,b), more pronounced on the left. An interscapular large segmented dark hyperpigmentation area with a verruccal epidermal nevus of about 20 × 10 cm and 15 × 3 cm was present (Figure 2c). The hyperkeratotic surface was partially fissured and the patches sharply bordered. Repeated admissions to clinical geneticists raised suspicion upon a genetic disorder and the tentative diagnosis of Neurofibromatosis type 1 was made.

Neurological examination showed mild talipes equinovarus and slow progressive incomplete paraparesis of the legs with increased muscle tonus and spasticity. There was a positive Babinski reflex (pyramidal sign) and a missing cremaster- and sphincter ani reflex. Cranial nerve dysfunction was not seen. Since there was chronic slow progressive weakness of finger extension of digits III-V of the left hand, grabbing is only possible with digit I and II. To date there is still progressive transversal spinal cord syndrome with spasticity, paraparesis and pain. The patient is wheel chair bound and only can stand upright with assistance. The patient has a proximal neuropathy and no evidence for a distal neuropathy, since electromyography analysis is normal. Recent whole body MRI and DXA showed osteopenia, although normal FGF23 and low vitamin D levels were present in the blood sample of this individual.

At age 4 after tumor removal histology was performed of skin (verruca), intraneural tissue and lipoma tissue.

## Materials and methods

### Immunohistochemistry

For routine diagnostics an H&E staining was performed on 4 μm thick sections from formalin-fixed, paraffin-embedded (FFPE) tissue. For immunohistochemical labelling sections from FFPE tissue were pretreated and incubated with primary antibodies against S-100-protein (DAKO, Hamburg, Germany, # Z 0311, 1:8000), epithelial membrane antigen (EMA, DAKO, # M 0613, 1:200) and neurofilament (DAKO, # M0762, 1:800) in an automated stainer (Ventana Medical Systems, Tucson, AZ, USA) according to standard protocols. As detection was the peroxidase method used with diaminobenzidine as chromogen (Ultraview DAB, 760–500, Ventana).

### Mutation analysis

The DNA was extracted from blood and three FFPE tissue samples (verrucous skin, intraneural perineurioma and lipoma) by use of standard techniques. All DNA samples were screened for the following *RAS* and *PIK3CA* mutations: c.37G > C (p.Gly13Arg) in *HRAS* (NM_005343.2) and c.35G > A (p.Gly12Asp) in *KRAS* (NM_004985.3) and c.1258 T > C (p.Cys420Arg), c.1624G > A (p.Glu542Lys) and c.3140A > G (p.His1047Arg) in *PIK3CA* (NM_006218.2). In total, 5 primer pairs were designed to maximum amplify the corresponding fragments of 300 bp (primer sequences available on request). All primers were tagged with M13 sequences to facilitate sequencing. The PCR products were bidirectionally Sanger sequenced and run on an ABI sequencer.

All procedures performed were in accordance with the Declaration of Helsinki.

## Results

Histological sections of the three samples showed a typical lipoma and skin with hyperplasia confirming verrucous epidermal hyperplasia. In the nerve tissue ganglion cells and spindle shaped cells arranged in concentric patterns were observed resembling a perineurioma or onion bulb formation, morphologically not consistent with a schwannoma or neurofibroma. There was no nuclear atypia and no mitoses.

**Figure 3:**
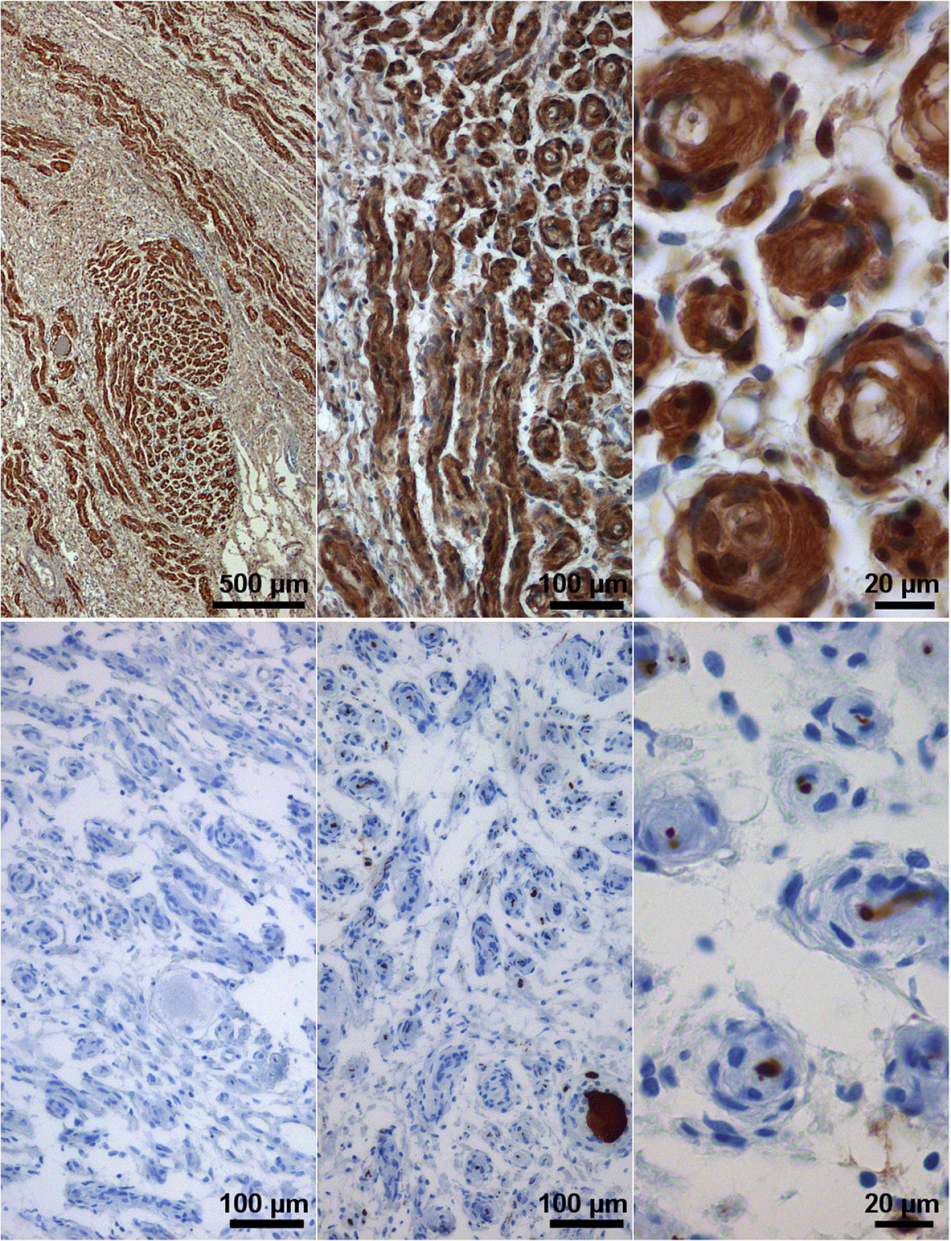
**Hypertrophic changes in a nerve root located within a conglomerate tumor.** Upper row, nerve root with hypertrophic nerve fascicles with extensive onion bulb formation by S-100-protein expressing schwann cells; lower row, left, lack of EMA expression in the tissue; middle and right, neurofilament detection in a neuron and axons in the center of onion bulbs.

Upon immunohistochemical labelling with antibodies against S100 protein, neurofilament and EMA, the onion bulb structures were strongly positive for S-100-protein (Figure 3 upper row) and commonly contained a neurofilament-positive central axon (Figure 3 lower row). No EMA expression was detected (Figure 3).

**Figure 4:**
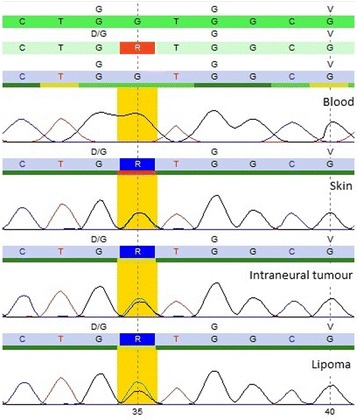
**Sanger sequences showing the c.35G > A (p.Gly12Asp) mutation (in yellow) in the** ***KRAS*** **gene in the skin sample, in the tumor sample with concentric intraneural Schwann cell proliferation and in the lipoma sample.** This mutation was not present in the blood sample from the patient.

Previously, neurofibromatosis type 1 was clinically and genetically ruled out. None of the specific mutations in the *HRAS* or *PIK3CA* genes were found in the DNA extracted from the three tissue samples. However, in all three samples the c.35G > A (p.Gly12Asp) missense mutation in the *KRAS* gene was detected. The c.35G > A (p.Gly12Asp) mutation in *KRAS* was in all three samples present in a heterozygous state (Figure 4), with an average allele frequency of 44% in the hyperpigmented verrucous skin sample, 49% in the tumor sample with the concentric intraneural Schwann cell proliferation and 56% in the lipoma sample based on peak height of both nucleotides at position 35. No c.35G > A (p.Gly12Asp) mutation in the *KRAS* gene was detected in DNA extracted from peripheral white blood cells from the patient.

## Discussion

The dermatological features of the reported patient are in accordance with the phenotype of previously described patients with KENS. The patient in this study presented with hyper- and hypopigmented regions and with verrucous hyperpigmented skin lesions. In addition a paravertebral conglomerate tumour was present next to the cervical and thoracic spine with a large intraspinal lipoma causing compression and atrophy of the spinal cord with progressive paraparesis. It is the first time that a myelopathy/proximal neuropathy in combination with the presence of a lipoma is reported in KENS. Further we could identify Schwann cell proliferation within the nerve resembling onion bulb formation, however, a typical schwannoma, as described by Bertola et al. 2012 in a patient with a germline *KRAS* (p.Lys5Glu) mutation [7], was not demonstrated. Recently epidermal nevus syndrome was described in a patient with an intra-spinal lipoma and cervical root neurofibromas [8]. In the same paper a further case had MRI changes suggestive for lumbo-sacral nerve root tumours which were asymptomatic. This patient was clinically diagnosed with CLOVES syndrome without co-occuring epidermal nevi.

We identified the same c.35G > A (p.Gly12Asp) *KRAS* mutation in the hyperpigmented verrucous skin tissue, intraneural perineurioma and lipoma. This mutation was not detected in the corresponding leukocytes of the same patient. The data confirm the diagnosis of KENS as a mosaic RASopathy. This mutation has not been reported as a germline mutation in individuals with Noonan or CFC syndrome and the clinical phenotype is distinctive compared to these syndromes. The c.35G > A (p.Gly12Asp) *KRAS* mutation has extensively been reported in tumour tissue described in the open access database COSMIC (Catalogue Of Somatic Mutations In Cancer) suggesting its strong oncogenic character. COSMIC contains information on publications, samples and mutations in cancer tissues. Codon 12 is the most frequently mutated codon of *KRAS* in malignancies. The c.35G > A (p.Gly12Asp) *KRAS* mutation has been identified frequently in cancers from colon, pancreas, lung, biliary tract and ovary.

To date, only 8 cases were diagnosed with KENS, molecularly characterized and their data are summarized in Table 1 [8-15]. Three males and 5 females were diagnosed with KENS and the age of presentation varied between newborn and 21 years. The epidermal nevus can be extensive, multiple epidermal nevi can be present or the nevus can be bounded by the ventral midline. The other clinical symptoms can differ between individuals and can potentially be very severe (brain abnormalities, benign and malignant tumours). Six different somatic mutations were identified in KENS with pathogenic mutations in the *KRAS* (c.35G > A), *HRAS* (c.34G > A, c.37G > C), *FGFR3* (c.742C > T, c.746C > G) and *PIK3CA* (c.3140A > T) genes. The epidermal nevus was molecularly investigated in 7/8 cases resulting in the identification of the responsible mutation. Leucocytes are often tested, but the mutation is frequently absent. Data for other tissues are variable as well as the percentage of mosaicism.

**Table 1:** **Clinical characteristics of patients with KENS in the literature**

**Case** ^**(reference)**^	**1 [** 9 **]**	**2 [** 10 **]**	**3 [** 11 **]**	**4 [** 12 **]**	**5 [** 13 **]**	**6 [** 14 **]**	**7 [** 15 **]**	**8 [** 8 **]**
**Age at presentation (years)**	Newborn	19	14	17	12	5	Newborn	21
**Sex**	F	M	M	F	F	F	F	M
**Clinical features**	EN following lines of Blaschko, rhabdomyosarcoma, micropolycystic kidneys and growth retardation	EN, urothelial-cell carcinoma of the bladder	Multiple EN, hypotonia of the right arm, thymoma, cystic lesions in hand and cervical bones	Systemic EN following the lines of Blaschko sharply bounded by ventral midline, scoliosis	Extensive EN and mild facial dysmorphy	Systematized EN, seizures, delayed language and psychomotor development, brain abnormalities	Verrucous hyperpigmented streaks sharply demarcated at midline, seizures and brain abnormalities (6 months)	Multiple EN, multiple spinal tumors, lipoma, duplic renal arteries, ectasia of aorta, scoliosis
**Gene mutation**	*KRAS*	*HRAS*	*HRAS*	*FGFR3*	*FGFR3*	*FGFR3*	*FGFR3*	*PIK3CA*
	c.35G > A (p.Gly12Asp)	c.34G > A (p.Gly12Ser)	c.37G > C (p.Gly13Arg)	c.742C > T (p.Arg248Cys)	c.742C > T (p.Arg248Cys)	c.742C > T (p.Arg248Cys)	c.746C > G (p.Ser249Cys)	c.3140A > T (p.His1047Leu)
**Tissue investigated:**								
**-carrying mutation**	Epidermal component EN, rhabodmyosarcoma	Epidermal nevus, 3 urothelial-cell carcinomas, lung metastasis, normal lung tissue, blood leukocytes and non-malignant urothelium	Both EN and thymoma	EN, oral mucosa and leucocytes	Verrucous EN	EN, blood leucocytes	EN, the unaffected skin, urothelial cells	Spinal tissue (neurofibromas)
**-mutation absent**	Dermal component EN, normal skin, blood	Bladder-muscle layer, 2 cutaneous angiomas	Lymphocytes	Hair roots, normal skin	Blood, buccal brushings, hair roots	Normal skin	Blood leucocytes	Dermis (posterior thoracic region)

EN: epidermal nevus; F: female; m: male.

## Conclusion

KENS patients are still young when the first potential complications occur. Therefore, it is important that patients are diagnosed as early as possible to provide a better multidisciplinary follow up and therapy when needed. If patients are diagnosed with epidermal nevi and a pathogenic *KRAS, PIK3CA* or *FGFR3* mutation, they should undergo further medical examination and potentially also further imaging (MRI) to identify the extent of disease. Mosaicism may delay diagnosis and complicate clinical management of such cases, as genetic confirmation of disease depends on appropriate tissue sampling.

## Consent

Written informed consent was obtained from the patient for publication of this Case report and any accompanying images. A copy of the written consent is available for review by the Editor of this journal. The study was carried out in compliance with the Helsinki Declaration and in accordance with the ethical requirements for case reports of the ethical board Hamburg.

## Abbrevations

ABI: Applied biosystems instruments
bp: Base pairs
c.37G > C (p.Gly13Arg): Nomenclature for the description of sequence variations
C3, 5, 7: Cervikal spinal segment 3, 5, 7
CFC: Cardiofaciocutaneous syndrome
CLOVES: Congenital lipomatous overgrowth, Vascular malformations, Epidermal nevi, Scoliosis/skeletal and spinal anomalies
Cm: Centimeter
COSMIC: Catalogue of somatic mutations in cancer
DNA: Deoxyribonucleic acid
DXA: Dual-energy X-ray absorptiometry
EMA: Epithelial membrane antigen
FFPE: Formalin-fixed, paraffin-embedded tissue
FGF23: Fibroblast growth factor 23
FGFR3: Fibroblast growth factor 3
g: Gramm
H&E: Hematoxylin and eosin stain
HRAS: HRas G-protein of the Ras subfamily
KEN: Keratinocytic epidermal nevus
KENS: Keratinocytic epidermal nevus syndrome
M13: DNA sequencing primers
MRI: Magnetic resonance imaging
PIK3CA: Phosphatidylinositol-4,5-bisphosphate 3-kinase, catalytic subunit alpha
RAS: ‘Rat sarcoma’
S-100: S-100 protein-family
T1: Thoracic spinal segment 1
T1/T2-weighted: T1/T2 relaxation time
Vitamin D: Cholecalciferol
